# Cancer associated fibroblasts (CAFs) are activated in cutaneous basal cell carcinoma and in the peritumoural skin

**DOI:** 10.1186/s12885-017-3663-0

**Published:** 2017-10-07

**Authors:** Silje Haukali Omland, Erika Elgstrand Wettergren, Tobias Mourier, Anders Johannes Hansen, Maria Asplund, Sarah Mollerup, Robert Robert

**Affiliations:** 1 Department of Dermato-Venerology, Bispebjerg University Hospital, Bispebjerg Bakke 23, 2400 Copenhagen, Nordvest Denmark; 2 Centre for GeoGenetics, Natural History Museum, University of Copenhagen, Copenhagen, Denmark; 3 Division of Dermatology, Faculty of Medicine, University of Alberta, Edmonton, Canada

## Abstract

**Background:**

Cutaneous basal cell carcinoma (BCC) is the commonest cancer worldwide. BCC is locally invasive and the surrounding stromal microenvironment is pivotal for tumourigenesis. Cancer associated fibroblasts (CAFs) in the microenvironment are essential for tumour growth in a variety of neoplasms but their role in BCC is poorly understood.

**Methods:**

Material included facial BCC and control skin from the peritumoural area and from the buttocks. With next-generation sequencing (NGS) we compared mRNA expression between BCC and peritumoural skin. qRT-PCR, immunohistochemical and immunofluorescent staining were performed to validate the NGS results and to investigate CAF-related cyto-and chemokines.

**Results:**

NGS revealed upregulation of 65 genes in BCC coding for extracellular matrix components pointing at CAF-related matrix remodeling. qRT-PCR showed increased mRNA expression of CAF markers FAP-α, PDGFR-β and prolyl-4-hydroxylase in BCC. Peritumoural skin (but not buttock skin) also exhibited high expression of PDGFR-β and prolyl-4-hydroxylase but not FAP-α. We found a similar pattern for the CAF-associated chemokines CCL17, CCL18, CCL22, CCL25, CXCL12 and IL6 with high expression in BCC and peritumoural skin but absence in buttock skin. Immunofluorescence revealed correlation between FAP-α and PDGFR-β and CXCL12 and CCL17.

**Conclusion:**

Matrix remodeling is the most prominent molecular feature of BCC. CAFs are present within BCC stroma and associated with increased expression of chemokines involved in tumour progression and immunosuppression (CXCL12, CCL17). Fibroblasts from chronically sun-exposed skin near tumours show gene expression patterns resembling that of CAFs, indicating that stromal fibroblasts in cancer-free surgical BCC margins exhibit a tumour promoting phenotype.

**Electronic supplementary material:**

The online version of this article (doi: 10.1186/s12885-017-3663-0) contains supplementary material, which is available to authorized users.

## Background

Basal cell carcinoma (BCC) of the skin is the most frequent cancer worldwide and the incidence is increasing [1]. BCC is locally invasive and the microenvironment surrounding BCC is crucial for the tumourigenesis, which was emphasised by auto-transplantation experiments where human BCC failed to grow in the absence of stroma [2]. In particular, fibroblasts in the surrounding tumour stroma seem essential for a variety of neoplasms [3, 4]. These cancer associated fibroblasts (CAFs) are characterised by a distinct activated phenotype and by expression of a variety of markers, such as fibroblast activated protein-α (FAP-α) and platelet-derived growth factor receptor β (PDGRF-β) [3]. Production of extracellular matrix components and cytokine secretion are some of the mechanism induced by CAF to promote tumour growth [5]. The role of CAFs has been widely investigated in breast cancer where they promote tumour growth via interaction with neoplastic cells [5]. Interestingly, not only CAFs within or immediately adjacent to tumour but also CAFs from cancer-free tissue adjacent to tumour exhibited tumour-promotion [6]. Cross-talk between BCC and CAFs and related matrix-remodeling is suggested [7] but this has not been extensively studied.

With this study we show, by the use of next-generation sequencing (NGS), that the most predominant genes expressed in BCC are involved in matrix remodeling. The findings of CAFs also in the peritumoural skin suggest CAFs to mediate an environment susceptible to skin cancer development and recurrence.

## Methods

### Material

The study was approved by the Danish Regional Ethics Committee, protocol number: H-4-2013-197 and the Danish Data Protection Agency, journal number: BBH-2014-008, I-Suite: 02675. All participants gave signed informed consent.

Material consisted of facial BCC and peritumoural skin obtained during Mohs surgery and 4 mm punch biopsies from the buttock collected at the Department of Dermatology, Bispebjerg University Hospital, Denmark. All BCCs were clinically nodular BCCs. The peritumoural skin was taken after complete removal (microscopically verified) of cancerous tissue. For qRT-PCR we included skin from 18 patients (Table 1) from BCC, peritumoural skin and buttock skin. Since the RNA yield was lower for the buttock skin, not all qRT-PCR analyses could be performed for this group. For each analysis, the exact number of buttock samples is mentioned in the figure legend. Material for immunohistochemical staining was taken from the same samples as the ones for qRT-PCR by splitting the material in two and embedding half for immunohistochemistry in Tissue-Tek (Sakura, Leiden, Netherlands) while immediately freezing the other half for qRT-PCR in −80 °C until analysis. For NGS we included BCC and peritumoural skin from additional four patients taken during Mohs surgery and snap frozen at −80 °C until analysis.

**Table 1:** Baseline characteristics of the included patients

Sample no	Age	Ethnic origin	Tumor size, mm	Anatomic localisation
1	70–74	Caucasian	6×6	Temporal region
6	65–69	Caucasian	6×14	Cheek
7	65–69	Caucasian	7×8	Glabella
8	50–54	Asian	4×4	Nose
10	45–49	Caucasian	6×6	Nose
13	45–49	Caucasian	16×18	Cheek
15	70–75	Caucasian	4×4	Nose
20	55–59	Caucasian	7×7	Nose
23	70–74	Caucasian	7×10	Nose
24	60–64	Caucasian	10×17	Temporal region
29	45–49	Caucasian	11×11	Nose
30	50–54	Caucasian	5×5	Nose
35	75–79	Caucasian	22×22	Temporal region
36	70–74	Caucasian	10×14	Forehead
37	70–74	Caucasian	10×10	Cheek
38	80–84	Caucasian	10×18	Eyebrow
39	70–74	Caucasian	8×8	Nose
41	55–59	Caucasian	8×11	Cheek

### Quantitative real-time PCR (qRT-PCR)

The frozen skin material (BCC, peritumoral skin and buttock skin) including both the dermal and epidermal part, that was frozen until qRT-PCR analysis, was thawed in RNA later (Sigma, St. Louis MO, USA) and minced into 1 mm pieces. The method was performed as described previously [8]. All analyses were run as triplicates. The primer-probes used were: GAPDH (HS02758991_g1), IL6 (HS00985639_m1), CD3 (HS00174158_m1), CXCL12 (HS03676656_mH), CXCR4 (HS00607978_s1), CCL11 (HS_00237013_m1), CCL17 (HS00171074_m1), CCL18 (HS00268113_m1), CCL22 (HS01574247_m1), CCL25 (HS00608373_m1), PDGFRβ (HS01019589_m1), FAP-α (HS00990806_m1), Collagen 11A (HS01097664_m1) and P4HA2 (HS00990001_m1). The data obtained from CC17, CCL18, and CCL22 analyses have previously been published together with data on T-regs [8]. Obtained data were analysed by the ∆∆CT method [9, 10] with GAPDH as housekeeping reference gene.

#### Immunohistochemistry and immunofluorescence

For immunohistochemical staining, 10 μm sections were cut from the embedded tissue blocks on a Microm HM560 cryostat and mounted on glass slides for immunohistochemical and immunofluorescent stainings. For 3,3′-Diaminobenzidine (DAB) (Dako, Glostrup, Denmark) stainings, the sections were fixed using acetone at -20^o^ C for 10 min. Endogenous peroxidase activity was quenched using 0.3% H_2_O_2_ (Merck, Millipore, Darmstadt, Germany) in PBS for 15 min at room temperature (RT) in the dark. The sections were blocked in 10% horse serum (Gibco, Fisher Scientific, New Zealand) and 1% bovine serum albumin (BSA) (Sigma, St. Louis MO, USA) in PBS for 1 h at RT and followed by incubation with the primary antibody (rabbit anti-FAP-α (1:100, LS-C313051, LSBio, Seattle, USA), mouse anti- CXCL12 (1:40, MAB350, R & D systems, Oxon, UK), rabbit anti-Collagen 11A (1:100, ab64883, Abcam, Cambridge, UK), rabbit anti-CXCR4 (1:400, AHP442, AbD Serotec, Oxford, UK), rabbit anti-IL6 (1:600, ab6672, Abcam), rabbit anti-PDGFRβ (1:100, LS-C312148, LSBio), mouse anti-CCL17 (1:80, LS-C198166, LSBio), mouse anti-CCL22 (1:100, MAB336, R & D systems) or rabbit anti-P4HA2 (1:80, LS-C91131, LSBio)) diluted in 1%BSA in PBS over night at 4 °C. Incubation with secondary antibody (goat anti-rabbit HRP (1:200, P0448, Dako), goat anti-mouse HRP (1:200, P0447, Dako)) diluted in 10% horse serum and 1% BSA in PBS for 40 min at RT was performed. DAB was added to visualise the staining and the sections were incubated with haematoxylin for 1 min followed by coverslipping with glycergel mounting medium (Dako).

For immunofluorescence (IF) stainings, sections were fixed and blocked as described above (peroxidase quenching step was omitted) followed by incubation with combinations of two primary antibodies (rabbit anti-FAP-α (1:200, LS-C313051, LSBio), mouse anti-CCL17 (1:80, LS-C198166, LSBio) PCGFR-β (1:100, LS-C312148, LSBio), CXCL12 (1:40, MAB350, R & D systems, Oxon, UK), CCL22 (1:100, MAB336, R & D systems)) diluted in blocking solution overnight at 4 °C. Incubation with secondary antibodies (goat anti-rabbit Alexa568 (1:500, A11036, Life Technologies) and/or goat anti-mouse Alexa488 (1:500, A11001, Life Technologies, Thermo Fischer Scientific)) diluted in 1% BSA in PBS was then performed for 1 h at RT in the dark. This was followed by incubation with 0.5 μg/ml 4′,6-Diamino-2-phenylindole (DAPI) (Sigma) and coverslipping with glycergel mounting medium.

#### Transcriptome sequencing

Large RNAs were extracted and isolated from the samples using the NucleoSpin® miRNA kit [11] following the protocol for purification of small and large RNA in separate fractions but skipping the DNA digestion step. The frozen tissue samples were cut into smaller pieces on dry ice, transferred to lysis buffer containing two stainless steel beads of 2–3 mm in diameter, and homogenised using the TissueLyser II (Quiagen, Hilden, Germany) prior to extraction. For each sample, 5 μl extract were DNase treated for 30 min at 37° with 1 μl TURBO DNase enzyme (ThermoFisher, www.thermofisher.com) in a total volume of 50 μl. The RNA was subsequently purified using the RNeasy MinElute Cleanup Kit (Quiagen). Libraries were prepared from 100 ng of RNA, using the ScriptSeq v2 RNA-Seq Library Preparation kit (Epicentre, Illumina, www.illumina.com), following the manufacturer’s guidelines, and with 12 cycles of PCR amplification. Paired-end sequencing of 100 base pairs was performed on the Illumina HiSeq 2000 platform, yielding more than 380 million paired-end sequence reads (Additional file 1: Table S1). Removal of adapters, quality trimming and merging of paired reads was carried out using open-source AdapterRemoval software [12]. Reads were mapped onto the human genome (assembly version hg19) using the RNA-seq aligner RNAstar [13]. Potential PCR duplicate reads were discarded using the rmdup function in samtools [14]. Whereas approximately 97% of all reads mapped to the human genome, duplicate reads constituted a significant fraction leaving only around 48 million unique reads (Additional file 1: Table S1). From this mapping, transcript abundance was estimated running FLUX CAPACITOR [15] provided with the GENCODE gene annotation [16]. The raw number of reads assigned to each transcript by FLUX CAPACITOR was used as input for EdgeR (Version 3.2, Bioconductor) [17].

#### Statistical analysis

Statistical analyses were performed by unpaired Students t-test (normal distributed data) or Mann-Whitney U test (non-normal distributed data) using GraphPad Prism 4 (GraphPad software Inc., CA, USA). Statistical significance was set at *P* < 0.05.

The NGS data were analysed following estimation of dispersions, and genes differentially expressed between BCC and peritumoural skin were tested using the ExactTest function in EdgeR.

## Results

### mRNA expression analysis with NGS identifies remodeling of extracellular matrix in BCC

Analysis of mRNA expression was investigated by RNA sequencing, revealing upregulation of 542 genes in BCC, for number of reads see Additional file 1: Table S1. Of these, 65 genes were coding for extracellular matrix components or enzymes involved in matrix metabolism such as metalloproteinases (Additional file 2: Table S2) like WNT1-inducible-signaling pathway protein 1 (WISP-1), extracellular matrix protein 1, and matrix metallopeptidases (European Nucleotide Archive (http://www.ebi.ac.uk/ena), accession PRJEB12664). The expression pattern reflected the hallmarks of matrix remodeling seen in other cancer types, such as overexpression of lysol oxidase-like 2 (LOXL2), fibronectin, proteoglycans, factors involved in epithelial to mesenchymal transition such as lymphoid enhancer binding factor (LEF) [18–20] and αVβ6-integrin and collagen types VI and XI not normally encountered in skin [21]. Overexpression of prolyl-4-hydroxylase (P4H) and (PDGFR-β) in BCC pointed at a high level of CAFs.

### Identification of CAFs in BCC and peritumoural skin

**Fig. 1:**
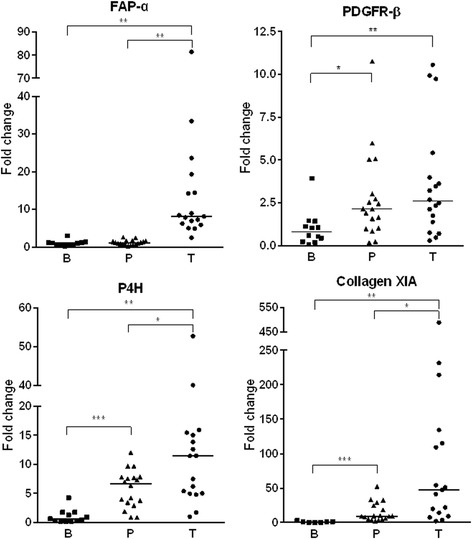
mRNA expression levels of CAF-markers and collagen XI in BCC, peritumoral skin and buttock skin. Gene expression of PDGRF-β, FAP-α, P4H and collagen XIA mRNA by qRT-PCR in BCC tumour (T), *n* = 18 (PDGFR-β, P4H), *n* = 17 (FAP-α, collagen XIA), peritumoural skin (P), n = 18, and buttock (B), *n* = 12 (PDGFR-β, P4H), *n* = 11 (FAP- α), *n* = 7 (collagen XIA). Significance level * = *p* < 0.5, ** = *p* < 0.001, *** = *p* < 0.0001. Where no *, no statiscally significant difference detected

CAFs are recognised by the expression of several markers including FAP-α, PDGFR-β and P4H [22, 23]. Genes from the latter two markers were found upregulated in our mRNA expression analysis. For validation of the NGS data and further investigation, the mRNA levels of FAP-α, PDGFR-β and P4H as well as collagen XIA in BCC, peritumoural skin and normal, non-UV exposed buttock skin were evaluated by the use of qRT-PCR. For most genes a consistent expression pattern was observed with the highest expression of CAF markers within BCC, followed by peritumoural skin and very low or no expression in the normal buttock skin (Fig. 1). FAP-α expression was only seen within BCC.

**Fig. 2:**
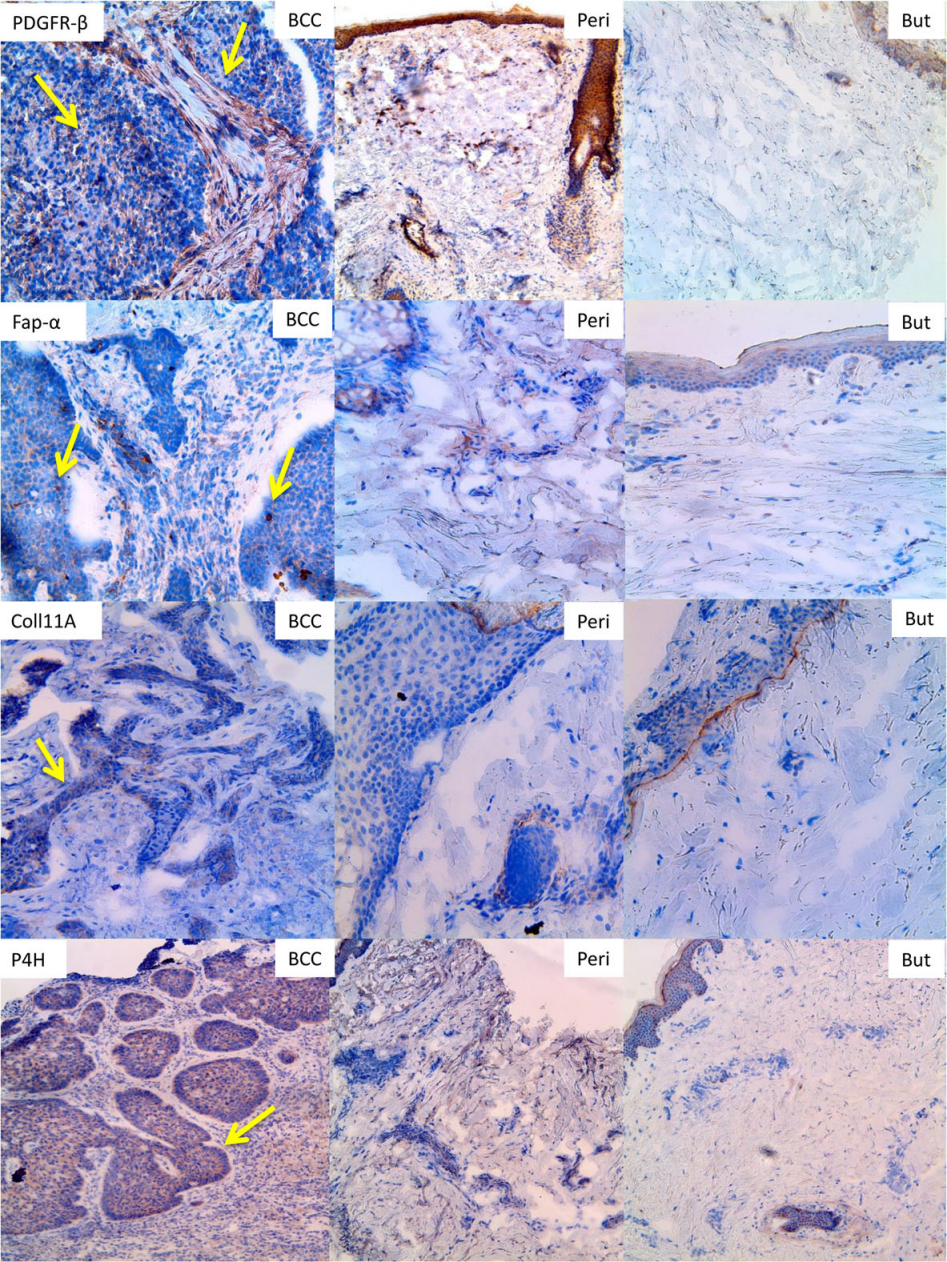
Immunohistochemical staining for CAF-markers and collagen XI. The photos show numerous positively stained cells in BCC, some in the peritumoural skin and few or none in the normal buttock skin of PDGRF-β, FAP-α, collagen XIA and (magnification ×20)

To identify the localisation of the CAFs we performed immunohistochemical staining for FAP-α, PDGFR-β, P4H and Collagen XIA. FAP-α, collagen XIA, P4H and PDGFR-β positive cells were highly abundant within the BCC tumour islands and in the near tumour periphery. Staining of the peritumoural skin also revealed FAP-α, collagen XIA, P4H and PDGFR-β positive cells, although not as numerous as in BCC. In the normal buttock skin, we identified a smaller amount of PDGFR-β and P4H positive cells in the epidermis but no collagen XIA or FAP-α positive cells (Fig. 2).

### CAFs are a source cytokines and chemokines involved in tumour progression and local immunosuppression

**Fig. 3:**
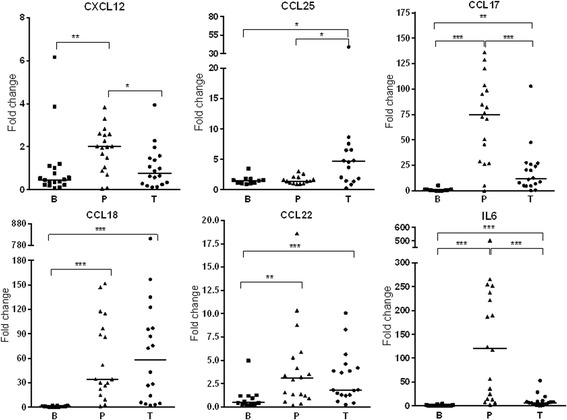
Gene expression of cytokine and chemokine mRNA by qRT-PCR. The mRNA expression levels of the cytokines CCL17, CCL18, CCL22, CCL25, and CXCL12 in BCC and peritumoural skin reveals increased expression in BCC and peritumoral skin whereas there is no expression in the normal non UV-exposed buttock skin. BCC tumour (T), n = 18 for all markers except CCL25 n = 17); peritumoural skin (P), n = 18 for all markers, and buttock (B), n = 18 (CXCL12), *n* = 16 (CCL17), *n* = 14 (CCL18), n = 12 (CCL22), n = 11 (CCL25). Significance level * = *p* < 0.5, ** = *p* < 0.001, *** = *p* < 0.0001. Where no *, no statiscally significant difference detected

Apart from the role in the synthesis of aberrant matrix components, CAFs can modulate the tumour microenvironment by producing chemokines and cytokines affecting the anti-tumour immune response [3, 7]. We performed qRT-PCR to detect the chemokine and cytokine mRNA levels in BCC, peritumoural-and buttock skin for the following genes: IL6, CXCL12, CCL17, CCL18, CCL22 and CCL25. These genes were selected since previous studies have pointed to them being involved in CAF-mediated tumour progression [6, 24, 25] or to the ability of these chemokines to attract regulatory T-cells (T-regs) [26–28]. For all cytokines and chemokines investigated, we found a high expression in BCC and/or peritumoural skin and barely any in the buttock skin (Fig. 3).

**Fig. 4:**
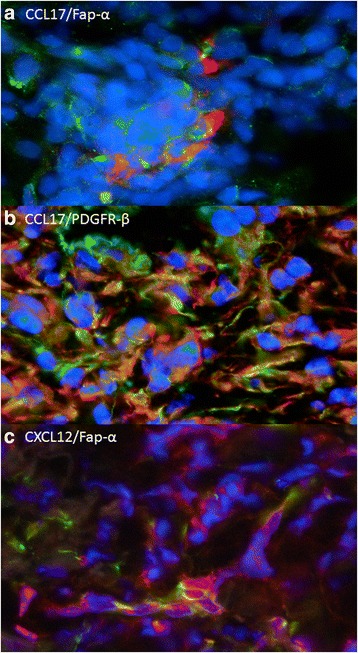
Immunofluorescent double staining of BCC with CAF-markers and chemokines. The staining suggests correlation between the CAF markers FAP-α and PDGFR-β and the chemokines CCL17 and CXCL12. **a** CCL17 (green)/FAP-α (red), **b** CCL17 (green)/PDGRF-β (red) (**c**) CXCL12 (green)/ FAP-α (red)

CXCL12, CCL17 and CCL22 play a role in the recruitment of T-regs to tumour sites and thereby inhibition of anti-tumour response [26–28]. We have previously shown an increased T-reg accumulation in relation to BCC^7^ and this led us to investigate a correlation between CAFs and T-reg attraction. IF double staining of FAP-α/CXCL12, FAP-α/CCL17, FAP-α/CCL22, as well as PDGFR-β/CCL17 and PDGFR-β/CCL22 were performed.The IF stainings indicated correlation between FAP-α/CXCL12 and FAP-α/CCL17 as well as PDGFR-β/CCL17 (Fig. 4), whereas no correlation was found between CCL22 and CAF markers. These findings support the hypothesis that CAFs mediate tumour progression by production of the T-reg chemotaxis CCL17 and CXCL12.

## Discussion

It is becoming evident that cancer development is dependent not only on neoplastic cells but is co-mediated by the tumour microenvironment [29]. For many cancer types, CAFs are essential contributors to tumour progression [3, 6]. The role of CAFs in BCC has not been investigated in detail. Lacina et al. suggested that fibroblasts from BCC play a regulatory role in BCC since they were capable of influencing growth and phenotype of normal cultured keratinocytes [30]. Our study provide further support to the concept that CAFs are involved in pathogenesis of BCC. We found increased expression of CAF-associated markers within BCC as well as in the peritumoural cancer-free tissue in contrast to absence of CAF-markers in the normal, non-UV exposed buttock skin. The mRNA expression of FAP-α was specific to BCC highlighting a pivotal role of FAP-α expressing CAFs in the development of BCC. The expression of PDGFR-β and P4H positive cells was found both within BCC as well as in the peritumoural cancer-free skin surrounding BCC. This indicates, as is the case for breast cancer [6], that fibroblasts in peritumoural skin exhibit a specific gene expression pattern intermediate between those of CAFs in BCC and normal fibroblasts (from buttock skin).

CAF-mediated tumour progression is exerted in many ways with chemokine and cytokine secretion being an essential contributor. In pancreatic cancer, CAF-induced IL6 secretion promotes cancer cell migration [24] and CAF-secreted CCL18 has been shown to promote tumour invasion in breast and ovarian cancer [25, 31]. Furthermore, CCL25 can mediate migration, invasion and matrix metalloproteinase expression in breast cancer cell lines [32]. With this study we found increased expression of CCL18 and CCL25 in BCC and peritumoural skin supporting a role for these chemokines in the pathogenesis of BCC. Increased IL-6 expression was found primarily in the peritumoural skin suggesting an impact of IL6 mainly in the peritumoural skin and a possible correlation to PDGRF-β and P4H, but not FAP-α expressing CAFs. This predominance of IL6 in the peritumoural surroundings is supported by a previously described role for IL-6 in mediating epithelial to mesenchymal transition [33].

Hindering the entry of T-cells into tumours or inactivating them could additionally be a CAF-mediated contributor to ineffective anti-tumour immune surveillance. CXCL12 is a chemokine causing direct immunosuppression via immobilisation of T-cells [32]. CXCL12 has been extensively studied and shown to be released by CAFs causing premalignant activities in tumour cells and in the cells of the tumour milieu [34, 35]. We found a high expression of CXCL12 in the peritumoural skin but low expression within BCC highlighting a role for CXCL12 primarily in the tumour microenvironment surrounding BCC. Accumulation of T-regs via CAF- secreted chemokines could also be orchestrated to avoid anti-tumour response. CXCL12, CCL22 and CCL17 have been described as critical factors for T-reg attraction in different cancer types [36, 37]. We have previously shown increased fraction of T-regs within BCC and in the peritumoural skin [8]. With this study, we demonstrate a possible correlation between CAFs and CXCL12 and CCL17 supporting the hypothesis of CAFs being involved in T-reg attraction. However, this possible correlation was demonstrated by IF only, and further studies are needed to understand mutual regulatory relationships between T-regs and CAFs.

Apart from increased CAF expression in relation to BCC, our study also demonstrated features of matrix remodeling with an important contributor being overexpression of collagen XI. Overexpression of this collagen has been shown to promote tumour progression and to be associated with poor outcome in many cancer types and is suggested as a target for future cancer therapy [21, 38]. To our knowledge, the presence of collagen XI in BCC has not previously been described. Our results clearly demonstrate increased collagen XI expression primarily within BCC but also in the peritumoural cancer-free tissue. In the normal buttock skin however, collagen XI expression was completely absent. Collagen XI has been assigned a metastatic potential for other cancer types [39]. BCC rarely metastasises and the abundance of this collagen within BCC might be an important mediator of local invasive growth. This, however, needs further investigation.

The tumour seed and soil hypothesis claims that for a tumour to grow, the microenvironment has to be susceptible [40]. Most reports claim CAFs to be initiated by tumour cells but there is evidence that CAFs are activated at an early stage contributing to tumour initiation [41]. The phenotype of the CAFs in the tumour-near skin intermediate between CAFs in BCC and fibroblasts in the normal skin could be mediated through secreted molecules able to diffuse from cancer cells through normal cells in the tumour periphery, without direct contact to the cancer cells [6]. The peritumoural skin in the present study was taken often >0.5 cm from the BCC rendering this mechanism unlikely. We hypothesise, that chronic UV-exposure partly mediates this induction of CAFs generating a tumour friendly microenvironment where neoplastic cells thrive. This could partly be caused by increased IL6 expression mediated by UV-exposure [42] since in cutaneous squamous cell carcinoma IL6 has been shown to be tumour-promoting by induction of CAFs [43]. In our study, we found a high expression of IL6 in the peritumoural skin, whereas IL6 was almost absent within BCC and completely absent in the non-UV-exposed buttock skin. This highly increased expression of IL6 in the tumour periphery might be a result of chronic UV exposure contributing to the shift in fibroblast phenotype. Regardless of whether CAFs in the peritumoural skin are induced by long-term UV-induced immunosuppression or by the neoplastic BCC cells, peritumoural CAFs could mediate an environment susceptible to skin cancer development or recurrence.

## Conclusion

In summary, we have shown features of matrix remodeling in BCC. Furthermore, we found that CAFs are abundant in BCC and may impact anti-tumour response by secretion of pro-carcinogenic cytokines and chemokines. Peritumoural skin also contains active, chemokine-secreting CAFs which could mediate an environment susceptible to skin cancer development or recurrence. However, further functional studies are needed to clarify the role of CAFs in BCC.

## Additional files

Additional file 1: Table S1. The number of reads in the mRNA sequencing analysis and the following reads that were actually mapped. Whereas approximately 97% of all reads mapped to the human genome, duplicate reads constituted a significant fraction leaving only around 48 million unique reads. (DOCX 33 kb)

Additional file 2: Table S2. The list contains the 65 genes coding for extracellular matrix components or enzymes involved in matrix metabolism that were found upregulated in BCC. (DOCX 18 kb)

## Abbreviations

BCC: Basal cell carcinoma
CAFs: Cancer associated fibroblasts
FAP-α: Fibroblast activated protein-α
NGS: Next-generation sequencing
P4H: Prolyl-4-hydroxylase
PDGRF-β: Platelet-derived growth factor receptor β
Regulatory T-cells: T-regs
