# Correction to: Cancer associated fibroblasts (CAFs) are activated in cutaneous basal cell carcinoma and in the peritumoural skin

**DOI:** 10.1186/s12885-018-3987-4

**Published:** 2018-01-30

**Authors:** Silje Haukali Omland, Erika Elgstrand Wettergren, Sarah Mollerup, Maria Asplund, Tobias Mourier, Anders Johannes Hansen, Robert Gniadecki

**Affiliations:** 1 Department of Dermato-Venerology, Bispebjerg University Hospital, Bispebjerg Bakke 23, 2400 Copenhagen, Nordvest Denmark; 2 Centre for GeoGenetics, Natural History Museum, University of Copenhagen, Copenhagen, Denmark; 3 Division of Dermatology, Faculty of Medicine, University of Alberta, Edmonton, Canada

## Correction

After publication of the original article [1] it was identified that order of the author list had been presented incorrectly. The author Robert Gniadecki’s surname was also incorrect in the original article.

The original article has been updated and the updated author list is also presented in this Correction.
