# Supplementary material for: Cancer associated fibroblasts (CAFs) are activated in cutaneous basal cell carcinoma and in the peritumoural skin
Source: BMC Cancer. 2017 Oct 7;17:675. doi: 10.1186/s12885-017-3663-0 (PMC5806272; doi:10.1186/s12885-017-3663-0)
Supplement: Supplementary file 1 — The number of reads in the mRNA sequencing analysis and the following reads that were actually mapped. Whereas approximately 97% of all reads mapped to the human genome, duplicate reads constituted a significant fraction leaving only around 48 million unique reads. (DOCX 33 kb) [file 12885_2017_3663_MOESM1_ESM.docx]

Supplementary Table 1.

Title: The number of reads in the transcriptome sequencing.

Legend: The number of reads in the mRNA sequencing analysis and the following reads that were actually mapped. Whereas approximately 97% of all reads mapped to the human genome, duplicate reads constituted a significant fraction leaving only around 48 million unique reads.

Abbreviation: BCC= basal cell carcinoma
