# Supplementary material for: Cancer associated fibroblasts (CAFs) are activated in cutaneous basal cell carcinoma and in the peritumoural skin
Source: BMC Cancer. 2017 Oct 7;17:675. doi: 10.1186/s12885-017-3663-0 (PMC5806272; doi:10.1186/s12885-017-3663-0)
Supplement: Supplementary file 2 — The list contains the 65 genes coding for extracellular matrix components or enzymes involved in matrix metabolism that were found upregulated in BCC. (DOCX 18 kb) [file 12885_2017_3663_MOESM2_ESM.docx]

Supplementary Table 2.

Titel: A selected list of genes found upregulated in BCC.

| 1. ADAM metallopeptidase domain 19 [Source:HGNC Symbol;Acc:HGNC:197] |
| --- |
| 2. ADAM metallopeptidase with thrombospondin type 1 motif, 12 [Source:HGNC Symbol;Acc:HGNC:14605] |
| 3. ADAM metallopeptidase with thrombospondin type 1 motif, 17 [Source:HGNC Symbol;Acc:HGNC:17109] |
| 4. ADAM metallopeptidase with thrombospondin type 1 motif, 2 [Source:HGNC Symbol;Acc:HGNC:218] |
| 5. ADAM metallopeptidase with thrombospondin type 1 motif, 3 [Source:HGNC Symbol;Acc:HGNC:219] |
| 6. archaelysin family metallopeptidase 2 [Source:HGNC Symbol;Acc:HGNC:28041] |
| 7. archaelysin family metallopeptidase 2 pseudogene 1 [Source:HGNC Symbol;Acc:HGNC:26491] |
| 8. cadherin 22, type 2 [Source:HGNC Symbol;Acc:HGNC:13251] |
| 9. cadherin 5, type 2 (vascular endothelium) [Source:HGNC Symbol;Acc:HGNC:1764] |
| 10. collagen, type I, alpha 1 [Source:HGNC Symbol;Acc:HGNC:2197] |
| 11. collagen, type I, alpha 2 [Source:HGNC Symbol;Acc:HGNC:2198] |
| 12. collagen, type III, alpha 1 [Source:HGNC Symbol;Acc:HGNC:2201] |
| 13. collagen, type IV, alpha 1 [Source:HGNC Symbol;Acc:HGNC:2202] |
| 14. collagen, type IV, alpha 2 [Source:HGNC Symbol;Acc:HGNC:2203] |
| 15. collagen, type V, alpha 1 [Source:HGNC Symbol;Acc:HGNC:2209] |
| 16. collagen, type VI, alpha 1 [Source:HGNC Symbol;Acc:HGNC:2211] |
| 17. collagen, type VI, alpha 3 [Source:HGNC Symbol;Acc:HGNC:2213] |
| 18. collagen, type VII, alpha 1 [Source:HGNC Symbol;Acc:HGNC:2214] |
| 19. collagen, type XI, alpha 1 [Source:HGNC Symbol;Acc:HGNC:2186] |
| 20. collagen, type XIV, alpha 1 [Source:HGNC Symbol;Acc:HGNC:2191] |
| 21. collagen, type XV, alpha 1 [Source:HGNC Symbol;Acc:HGNC:2192] |
| 22. collagen, type XVI, alpha 1 [Source:HGNC Symbol;Acc:HGNC:2193] |
| 23. elastin [Source:HGNC Symbol;Acc:HGNC:3327] |
| 24. extracellular matrix protein 1 [Source:HGNC Symbol;Acc:HGNC:3153] |
| 25. fibrillin 3 [Source:HGNC Symbol;Acc:HGNC:18794] |
| 26. fibronectin 1 [Source:HGNC Symbol;Acc:HGNC:3778] |
| 27. fibronectin type III domain containing 1 [Source:HGNC Symbol;Acc:HGNC:21184] |
| 28. fibrosin-like 1 [Source:HGNC Symbol;Acc:HGNC:29308] |
| 29. Fraser extracellular matrix complex subunit 1 [Source:HGNC Symbol;Acc:HGNC:19185] |
| 30. heparan sulfate proteoglycan 2 [Source:HGNC Symbol;Acc:HGNC:5273] |
| 31. hepatocyte growth factor-regulated tyrosine kinase substrate [Source:HGNC Symbol;Acc:HGNC:4897] |
| 32. hyaluronan and proteoglycan link protein 1 [Source:HGNC Symbol;Acc:HGNC:2380] |
| 33. insulin-like growth factor 2 mRNA binding protein 2 [Source:HGNC Symbol;Acc:HGNC:28867] |
| 34. integrin, alpha 11 [Source:HGNC Symbol;Acc:HGNC:6136] |
| 35. integrin, alpha 4 (antigen CD49D, alpha 4 subunit of VLA-4 receptor) [Source:HGNC Symbol;Acc:HGNC:6140] |
| 36. integrin, beta 6 [Source:HGNC Symbol;Acc:HGNC:6161] |
| 37. integrin, beta-like 1 (with EGF-like repeat domains) [Source:HGNC Symbol;Acc:HGNC:6164] |
| 38. laminin, alpha 2 [Source:HGNC Symbol;Acc:HGNC:6482] |
| 39. laminin, alpha 4 [Source:HGNC Symbol;Acc:HGNC:6484] |
| 40. laminin, alpha 5 [Source:HGNC Symbol;Acc:HGNC:6485] |
| 41. latent transforming growth factor beta binding protein 1 [Source:HGNC Symbol;Acc:HGNC:6714] |
| 42. latent transforming growth factor beta binding protein 4 [Source:HGNC Symbol;Acc:HGNC:6717] |
| 43. leucine proline-enriched proteoglycan (leprecan) 1 [Source:HGNC Symbol;Acc:HGNC:19316] |
| 44. lysyl oxidase-like 2 [Source:HGNC Symbol;Acc:HGNC:6666] |
| 45. matrix metallopeptidase 11 (stromelysin 3) [Source:HGNC Symbol;Acc:HGNC:7157] |
| 46. matrix metallopeptidase 13 (collagenase 3) [Source:HGNC Symbol;Acc:HGNC:7159] |
| 47. membrane metallo-endopeptidase [Source:HGNC Symbol;Acc:HGNC:7154] |
| 48. metallophosphoesterase domain containing 1 [Source:HGNC Symbol;Acc:HGNC:1306] |
| 49. nephronectin [Source:HGNC Symbol;Acc:HGNC:27405] |
| 50. nidogen 1 [Source:HGNC Symbol;Acc:HGNC:7821] |
| 51. nidogen 2 (osteonidogen) [Source:HGNC Symbol;Acc:HGNC:13389] |
| 52. p21 protein (Cdc42/Rac)-activated kinase 1 [Source:HGNC Symbol;Acc:HGNC:8590] |
| 53. platelet-derived growth factor receptor, beta polypeptide [Source:HGNC Symbol;Acc:HGNC:8804] |
| 54. prolyl 4-hydroxylase, alpha polypeptide II [Source:HGNC Symbol;Acc:HGNC:8547] |
| 55. protocadherin 11 X-linked [Source:HGNC Symbol;Acc:HGNC:8656] |
| 56. protocadherin beta 12 [Source:HGNC Symbol;Acc:HGNC:8683] |
| 57. raftlin, lipid raft linker 1 [Source:HGNC Symbol;Acc:HGNC:30278] |
| 58. Rho GTPase activating protein 25 [Source:HGNC Symbol;Acc:HGNC:28951] |
| 59. Rho GTPase activating protein 33 [Source:HGNC Symbol;Acc:HGNC:23085] |
| 60. Rho guanine nucleotide exchange factor (GEF) 11 [Source:HGNC Symbol;Acc:HGNC:14580] |
| 61. Rho-associated, coiled-coil containing protein kinase 1 pseudogene 1 [Source:HGNC Symbol;Acc:HGNC:37832] |
| 62. secreted protein, acidic, cysteine-rich (osteonectin) [Source:HGNC Symbol;Acc:HGNC:11219] |
| 63. trio Rho guanine nucleotide exchange factor [Source:HGNC Symbol;Acc:HGNC:12303] |
| 64. wingless-type MMTV integration site family, member 10A [Source:HGNC Symbol;Acc:HGNC:13829] |
| 65. WNT1 inducible signaling pathway protein 1 [Source:HGNC Symbol;Acc:HGNC:12769] |
|  |
|  |

Legend: The list contains the 65 genes coding for extracellular matrix components or enzymes involved in matrix metabolism that were found upregulated in BCC.
